# Association of Host and Microbial Species Diversity across Spatial Scales in Desert Rodent Communities

**DOI:** 10.1371/journal.pone.0109677

**Published:** 2014-10-24

**Authors:** Yoni Gavish, Hadar Kedem, Irit Messika, Carmit Cohen, Evelyn Toh, Daniel Munro, Qunfeng Dong, Clay Fuqua, Keith Clay, Hadas Hawlena

**Affiliations:** 1 Department of Life Sciences, Ben Gurion University of the Negev, Beer Sheva, Israel; 2 Department of Microbiology and Immunology, Indiana University School of Medicine, Indianapolis, IN, United States of America; 3 Department of Biology, University of North Texas, Denton, TX, United States of America; 4 Department of Computer Science and Engineering, University of North Texas, Denton, TX, United States of America; 5 Department of Biology, Indiana University, Bloomington, IN, United States of America; University of Wisconsin-Madison, United States of America

## Abstract

Relationships between host and microbial diversity have important ecological and applied implications. Theory predicts that these relationships will depend on the spatio-temporal scale of the analysis and the niche breadth of the organisms in question, but representative data on host-microbial community assemblage in nature is lacking. We employed a natural gradient of rodent species richness and quantified bacterial communities in rodent blood at several hierarchical spatial scales to test the hypothesis that associations between host and microbial species diversity will be positive in communities dominated by organisms with broad niches sampled at large scales. Following pyrosequencing of rodent blood samples, bacterial communities were found to be comprised primarily of broad niche lineages. These communities exhibited positive correlations between host diversity, microbial diversity and the likelihood for rare pathogens at the regional scale but not at finer scales. These findings demonstrate how microbial diversity is affected by host diversity at different spatial scales and suggest that the relationships between host diversity and overall disease risk are not always negative, as the dilution hypothesis predicts.

## Introduction

Ecologists have been investigating the relationship between habitat heterogeneity and species diversity for more than 50 years. The occurrence of positive relationships between the two is widespread [1]–[5]. Nevertheless, recent theoretical and empirical syntheses refute the common wisdom that those relationships should always be positive and call for more detailed knowledge of the community structure, including the niche breadth of organisms and the order in which communities assemble in nature [2], [4], [6].

The gap between theory and our knowledge of natural communities is emphasized by communities of vertebrates and their associated microbes. The relationships between vertebrate and microbial diversity is of particular interest. Vertebrate-associated microbial communities exhibit a high level of complexity where microbes span different organizational levels including the host tissue, organ, the individual host, the host population and the host community [7], [8]. Likewise, host-microbial diversity associations can be analyzed over finer scales from individual hosts up to larger scales over geographical regions. Finally, host-associated microbes are involved in various relationships with their hosts including parasitism, commensalism and mutualism. From an applied perspective, the relationships between vertebrate hosts and their microbes may have important consequences for overall disease risk to humans and wild animals because the proportion of pathogens in the microbial community may vary with host diversity. Nonetheless, although natural communities of non-primate vertebrate host-associated microbes have been explored before [9]–[19], their community structure and the relationships between host and microbial diversity across scales remain obscure.

Increased host species diversity may impose two opposite effects on microbial species diversity resulting in two alternative hypotheses for the host-microbial diversity relationship. On one hand, it may be more difficult for microbes to invade and persist in diverse host communities because highly suitable host species become diluted among other, less suitable, host species. Therefore, the likelihood of stochastic microbe extinction increases with host diversity (equivalent to the area–heterogeneity tradeoff of free-living organisms [20]). On the other hand, microbe and host diversity may be positively correlated because higher host heterogeneity may allow a larger number of microbial species to coexist (equivalent to niche theory of free-living organisms [21]).

The overall relationship between host and microbial diversity may depend on the community structure of hosts and their associated microbes and the spatio-temporal scale of the analysis, as demonstrated by the relationship between habitat heterogeneity and species diversity of free-living organisms [4], [22], [23]. Because the likelihood of stochastic extinction is expected to decrease with increasing niche breadth of the microbial species [24], a community dominated by microbes with broader niches is less limited by suitable hosts and should exhibit a positive response to host diversity compared to a community dominated by microbes with narrower niches (see equivalent prediction for free-living organisms [20]). Likewise, at fine spatial scales, species distribution and coexistence may be governed by stochastic processes due to limited population sizes [25] and be less sensitive to changes in host diversity, while at larger spatial scales a significant portion of species would reach their niche requirements within a region [26]. Therefore, microbial communities at larger scales should exhibit a positive response to host diversity compared to communities at finer spatial scales.

**Figure 1:**
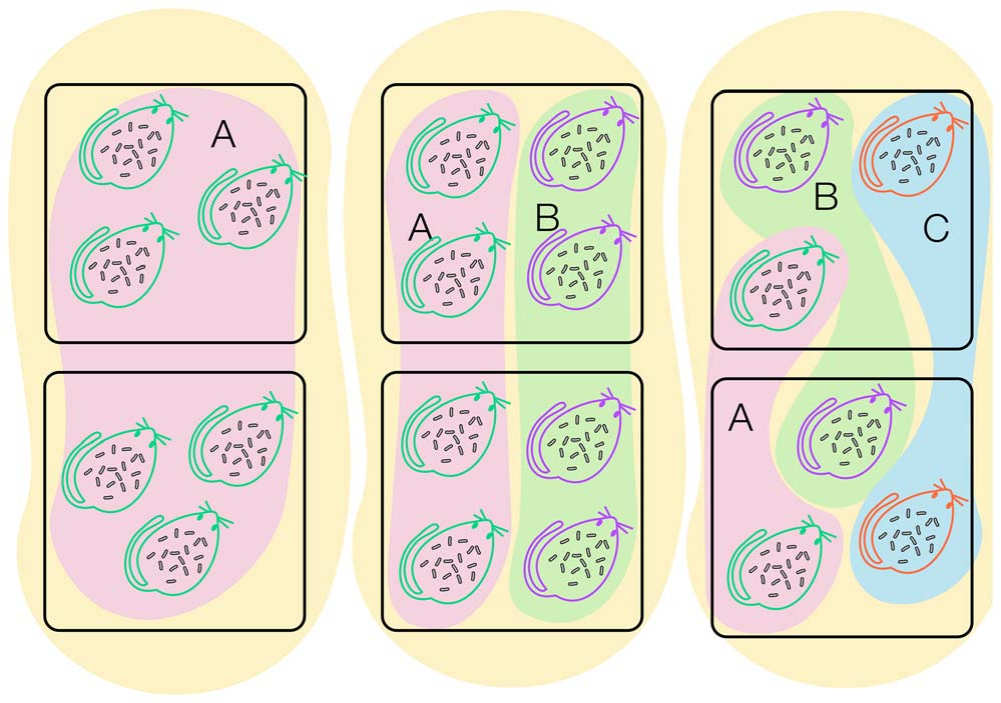
A schematic illustration of the experimental design. Rodents were trapped in 1-ha plots (squares), representing populations of *Gerbillus andersoni* [(A), host individuals are represented as green rodent outlines and the populations are shaded pink], *G. pyramidum* [(B), purple outlines and green shading] and *G. gerbillus* [(C), red outlines and blue shading]. The rodent populations were part of three communities; a single host species (*G. andersoni*, left tan shading), two (*G. andersoni* and *G. pyramidum*, middle tan shading) or three rodent species communities (right tan shading). Bacterial lineage (rod-like shapes) diversity and composition were assessed for three organization levels (individuals, populations and communities of hosts) nested within three hierarchical spatial scales (a host individual, a plot, and a region).

Accordingly, we employed a natural gradient of rodent species richness and investigated the structure of bacterial communities found in the rodent blood at several organizational levels nested in hierarchical spatial scales (Fig. 1). We tested two alternative hypotheses. The ‘negative host-microbial diversity relationship’ hypothesis assumes that microbes have relatively narrow niches and predicts that microbial diversity would decrease with increased host diversity. The alternative, ‘positive host-microbial diversity relationship’, hypothesis assumes that microbes have relatively broad niches and predicts that microbe and host diversity would be positively correlated. In addition, we hypothesized that at larger spatial scales, microbial diversity would be more likely to show positive response to host diversity than at finer scales.

Bacterial communities in vertebrate blood are convenient models to investigate the relationships between host species richness and bacterial community structure. While blood microbiome studies have been restricted to humans and represent only about 1% of all microbiomes examined [27], recent evidence suggests that a blood microbiome does exist and that it is comprised of translocated bacteria [28] and bacteria that penetrate the blood directly from the external environment [29] or via arthropod vectors [30]. Thus, the blood reflects the diversity of bacteria that the host is exposed to, including rare pathogens. At the same time, the blood microbiota is filtered and affected by host hormones and the circulating immune response and as such should be primarily subjected to the different selection pressures exerted by variation in host species diversity and less influenced by local environmental conditions and diet. Our data demonstrate that bacterial communities in rodent blood are comprised mostly of rare lineages and lineages which infect multiple rodent species. The composition and diversity of those communities at fine scales is primarily associated with the host species and not with host diversity. However, as predicted, at larger scales, bacterial diversity is positively associated with host species diversity, which suggests that the increased microbial diversity with increasing host diversity may raise overall disease risk.

## Material and Methods

### Experimental design and rodent trapping

The research was conducted in the dune ecosystem of the Western Negev desert (34°30′E and 30°55′N). We used the dune's natural field setting, in which clearly demarcated sites are populated with rodent communities of different species numbers in close proximity to one another. The three most common are single-species communities dominated by *Gerbillus andersoni* individuals, two-species communities composed of *G. andersoni* and *G. pyramidum* rodents and three-species communities composed of *G. andersoni*, *G. pyramidum* and *G. gerbillus* [31]. There are four other rodent species that may occur in those communities, but they are relatively rare. This study system is therefore ideal for testing host-microbial diversity relationships. While the gradient of host species richness is limited to three, all hosts belong to the same genus and co-occur in the same macro-habitats, minimizing abiotic and biotic heterogeneity that is not directly related to host species richness. Based on species composition records from previous rodent studies in the same area, we located 34 plots; 13, 14 and 7 plots of single-species, two-species and three-species communities, respectively. Note that three-species communities are relatively rare in the Negev desert and therefore were represented by a smaller number of plots.

Rodent trapping was conducted between July–August 2011 so that seasonal changes in conditions between trapping nights were not a confounding factor. To maintain independence and to avoid confounding time and treatments, all plots were located at least 40 m apart from each other [32] and pairs of single-host and multiple-host plots were sampled during the same trapping sessions. During each trapping session, rodents were captured in four or six 1-ha plots on three consecutive nights. Each captured individual was ear-tagged, identified according to species and sex, and weighed. Throughout the sampling period, 212 rodent individuals were tagged and no movement of any of these individuals was detected between plots, further emphasizing the independence of the plots.

We obtained 100 µL blood samples from a total of 90 rodent individuals of the three focal rodent species (Supplementary Methods in file S1). The trapping protocol was approved by the Ben Gurion University Committee for the Ethical Care and Use of Animals in Experiments (# IL-14-03-2011) and by the Nature and National Parks Protection Authority (# 2011/38146).

### Characterization of bacterial communities in rodent blood

DNA was extracted from rodent blood using a MoBio Bacteremia DNA isolation kit. We added 50 µl of blood to the Microbead tube and followed the manufacturer's instructions. We chose this kit and protocol since it gave the best results for spiked blood with known bacteria quantities. In each extraction session, a negative control was added in which all of the reagents were added to phosphate-buffered saline instead of blood. All negative controls were later subjected to pyrosequencing and were used to distinguish between blood-borne bacteria and contaminants. PCR, sequencing and the bioinformatics protocols were detailed in Hawlena et al. [33] and are also briefly described in Supplementary Methods in file S1.

For the bioinformatics analysis, pyrosequencing reads were processed using the Mothur package (version 1.22.0), as described in Schloss et al. [34]. Briefly, bacterial sequences were assigned to specific blood samples only if they perfectly matched the primer barcode sequence. The primer and barcode sequences were trimmed from the remaining sequences and the trimmed sequences with length less than 200 bp were removed. An additional denoise step was performed to remove low quality sequences, followed by a chimera detection and elimination. Sequences were binned into phylotypes where a phylotype (hereafter, lineage) is a group of microbes commonly defined by the level of sequence similarity between small subunit (16S) rRNA genes (e.g., ≥97% for a ‘species’-level lineage [35]). Accordingly, if two sequences were greater than or equal to 97% identity over their overlap regions, they were grouped into a single lineage. Classification to the species level was done by randomly sampling 100 member sequences for each lineage and comparing them to the RDP 10.26 database using BlastN [36] with an E-value cutoff of 10^−20^ [37]. Only lineages that had significantly higher relative abundance (≥2% of the bacterial community in any one sample) in the blood samples than in the negative controls were included in the analyses, following Hawlena et al. [33].

All sequences were deposited at the SRA site (study accession SRP044275; http://www.ncbi.nlm.nih.gov/Traces/sra/?study=SRP044275) and at Dryad, in addition to a metadata file that lists the barcode/primer, host species/sex and community species composition of each sample (http://datadryad.org/review?wfID=31217&token=e42a2295-10d6-4e20-ae3d-a7b06d6149d8; DOI is doi:10.5061/dryad.gh22m).

### Statistical analysis

The natural distribution of rodent species within our study site allowed exploration of the bacterial diversity and community composition at several organization levels nested within hierarchical spatial scales (Fig. 1). A single host individual forms the first level of organization at the smallest spatial scale. Then, we aggregated the raw lineage data over all host individuals from a given host species within a given plot. This yielded the raw data for the population level analysis at the plot scale. Further aggregation of all host individuals from all host species in a given plot yielded the community organization level at the plot scale. Next, we pooled the data for all host individuals from a given host species, yielding the raw data for the population level at the regional scale. Similarly, we have pooled the data from all host individuals over all host species to provide the raw data for the community level at the regional scale.

We quantified bacterial lineage diversity at each combination of organization level and scale using Fisher alpha diversity index, a reliable index of species diversity that is independent of sample size [38]. Bacterial community composition was assessed using the binary form of Bray-Curtis dissimilarity index [39]. We used presence/absence rather than abundance data to reduce the weight of possible pyrosequencing errors in abundance estimations [40]. Because lineage composition may also be affected by the total number of sequences, we normalized the number of sequences in each sample before analysis (Supplementary Methods in file S1). Detailed description of the quantification of bacterial lineage diversity and composition is provided in Supplementary Methods in file S1.

The overall contribution of individual lineages within a community to observed differences was explored using SIMPER, an analysis tool within PRIMER-E, where only bacteria that contributed consistently to the distinction between communities (i.e., when the ratio between the average and the standard deviation of dissimilarity between two lineages was greater than one) were regarded as good discriminating lineages.

The Generalized Linear Models (GLM) and non-parametric multivariate approaches could not be applied for the regional scale due to the limited sample size. To complement the qualitative presentation of host diversity on bacterial diversity at this scale, we performed non-linear regressions of Ranked Species Occupancy Curves (RSOC) and competed among six regression models, which are used to characterize the rank occupancy relationships of most ecological communities, following Jenkins (Supplementary Methods in file S1) [41].

To (i) quantify the likelihood for rare human pathogens and to (ii) assess the niche breadth of bacterial lineages in the three rodent communities, we first prepared lists of potential zoonotic, vector-borne human pathogens, host-specific and host opportunistic lineages and then estimated the proportion of zoonotic candidates and host-opportunistic lineages in each of the rodent communities. The list of potential zoonotic, vector-borne, human pathogens was based on the BlastN classification of the lineages (Table 1). It is possible that some of the listed organisms are not vector-borne zoonotic pathogens since we have not isolated and tested the pathogenicity and transmission dynamics of each organism. However, since we had created the list of potential pathogens before we looked for their presence in the different plot types, the list should not be biased toward one host community or the other. We characterized the niche breadth for only those bacterial lineages that were detected in at least three host individuals in the multiple-host species plots. A host-specific lineage was defined as one that was detected in a single host species in at least 90% of the positive blood samples for that lineage, while a host-opportunist lineage was associated with a single host species in less than 90% of the positive samples.

**Table 1:** Bacterial lineages suspected to be zoonotic pathogens and the host species and rodent community in which they were detected.

Best match	# of rodent species in a community	Host species	Indications for pathogenicity to humans
*Bartonella schoenbuchensis*	2	*G. andersoni*	Potential role in the etiology of deer ked dermatitis, which is pathogenic to humans [52]
*Streptococcus* sp.	2 & 3	*G. andersoni* and *G. gerbillus*	Many species are pathogenic for humans [53]
*Capnocytophaga canimorsus* (4 lineages, the one detected in the three rodent communities is indicated as *Capnocytophaga* 1 in Fig. 3)	3	*G. gerbillus*	Generally has low virulence in healthy individuals, but has been observed to cause severe illness in persons with pre-existing conditions [54]
		*G. pyramidum*	
	1 & 2 & 3	In all three species	
*Capnocytophaga* sp. S12–14	2	*G. pyramidum*	
*Corynebacterium urealyticum* (2 lineages)	3	*G. pyramidum*	A cause of urinary tract infection and encrusting cystitis or pyelitis [55]
*Finegoldia magna*	3	*G. gerbillus*	Is often associated with true infection [56]
*Helicobacter* sp. hokurin-1	3	*G. pyramidum*	Non-*pylori Helicobacter* species have been detected in human clinical specimens and are associated with chronic hepatobiliary and intestinal diseases in humans [57]
*Neisseria mucosa*	3	*G. andersoni*	Can cause endocarditis [58]
Uncultured *Leptotrichia* sp.	3	*G. pyramidum*	Have been recovered from patients with endocarditis [59]

Best match: Bacterial classification from known sequences that best matched the sample sequence (highest bit score).

### Accession Numbers

All sequences were deposited at the SRA site (study accession SRP044275) and at Dryad, in addition to metadata file that lists the barcode/primer, host species/sex and community species composition of each sample (can be viewed at http://datadryad.org/review?wfID=31217&token=e42a2295-10d6-4e20-ae3d-a7b06d6149d8; doi:10.5061/dryad.gh22m).

## Results

### Field samples and their microbiome

In total, 36 blood samples from 11 single-species (*G. andersoni*) plots, 37 samples from 10 two-species plots (19 from *G. andersoni* and 18 from *G. pyramidum*), and 17 samples from five three-species plots (five from *G. andersoni*, five from *G. pyramidum*, and seven from *G. gerbillus*) were successfully subjected to pyrosequencing. Number of total sequences per sample ranged from 35 to 353, with a mean of 161 and a mode of 64. These low numbers suggest that, similar to the bloodstream in healthy humans, the bloodstream in healthy rodents is a sterile environment compared to the gut or skin microbiomes [42], [43]. From those 90 DNA extracts, 161 lineages were significantly more abundant than the negative controls and represented at least 2% of the total number of sequences detected in any blood sample. The 161 lineages represented 73 bacterial genera (Table S1 in file S1). To our knowledge, this study provides the first record of *Cardinum* spp. and *Wolbachia* spp. from vertebrate hosts.

### Community structure of rodent-associated bacteria

Bacterial communities were comprised primarily of rare lineages, with 128 lineages out of the 161 lineages infecting a single host individual and 13 lineages infecting two host individuals (Fig. S1 in file S1). In the multi-species plots, from the 15 lineages that appeared in three host individuals or more, 13 showed no distinct preference (less than 90% prevalence) to any host species. The two lineages that showed very clear preference to a given host species were *Cardinium hertigii*-like bacterium, whose three occurrences were only from *G. pyramidum* individuals, and *Mycoplasma haemomuris*-like bacterium which showed a distinct preference for *G. andersoni*. Nonetheless, some lineages with less extreme host preference did contribute to distinguishing between host species (SIMPER analysis below). *M. haemomuris*-like bacterium had the highest overall prevalence, occurring in more than 50% of the blood samples.

### Determinants of lineage composition and diversity in bacterial communities at the individual host scale

**Figure 2:**
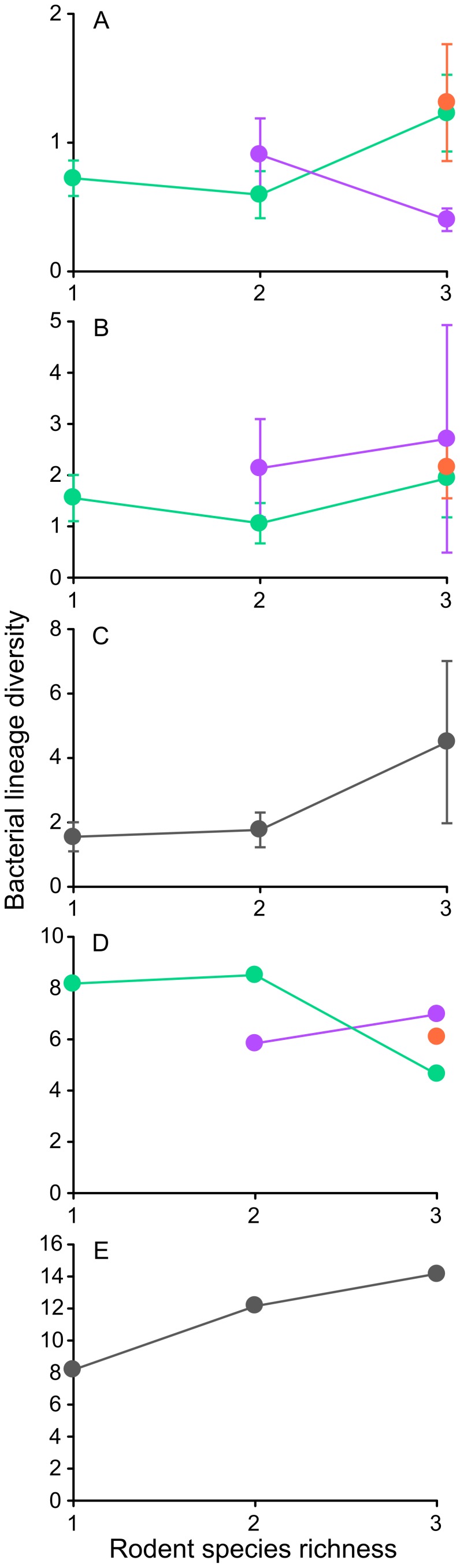
Relationships between bacterial lineage diversity (means ± SE of Fisher alpha) and rodent species richness. Relationships are quantified at different scales and for different organization levels. A- in individual hosts, B- at the plot scale in a population of hosts, C- at the plot scale in a community of hosts, D- at the regional scale in a population of hosts, E- at the regional scale in a community of hosts. The relationships between bacterial diversity and host diversity in individual hosts and host populations are illustrated separately for each host species (G*erbillus andersoni* is indicated in green, *G. pyramidum* in purple and *G. gerbillus* in red).

GLM analysis of bacterial diversity within individual hosts revealed a significant host species by host diversity interaction, suggesting that at this small scale, host diversity is an important determinant of bacterial diversity, although the direction of effect depended on the host species (Table 2). In particular, the presence of *G. gerbillus* in the rodent communities was positively associated with lineage diversity of bacterial communities in *G. andersoni* hosts and was negatively associated with lineage diversity of bacterial communities in *G. pyramidum* hosts (Fig. 2A).

**Table 2:** Statistical results testing the effects of host species, host diversity and their interaction on diversity measures of bacterial communities of individual rodents, rodent populations, and rodent communities.

Scale	Level of Host organization	Dependent variable	Statistical results: p (% = percentage of variance explained)		
			Host species	Host diversity	Interaction
Host Individual	Individual	Lineage diversity	NS	NS	P<0.05
		Lineage composition	P<0.0005 (15%)	p<0.05 (3%)	p<0.05 (2%)
Plot	Population	Lineage diversity	NS	NS	NS
		Lineage composition	P<0.0005 (10%)	NS	NS
	Community	Lineage diversity	NA	NS	NA
		Lineage composition	NA	NS	NA
Region	Community	Lineage prevalence	NA	* See RSOC results below	NA

Lineage diversity and composition were quantified at three spatial scales: Within host individuals, within 1-ha plots and within a region.

NS- No significant effects; NA- Not applicable.

*As the conventional statistical approaches could not be applied for the regional scale data due to the limited sample size, we performed non-linear regressions of ranked bacterial lineage occupancy curves (RSOC) and competed among six regression models.

The RSOC is changed from an asymmetric sigmoidal in single-species to exponential concave in multiple-species communities; see Table 3 and Fig. S3 in file S1 for quantitative results.

**Figure 3:**
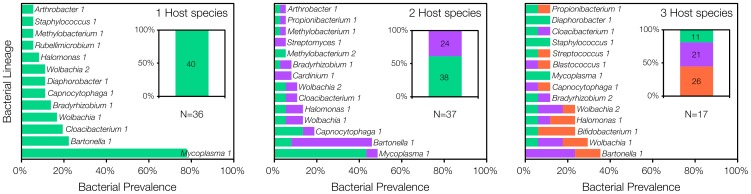
Bacterial prevalence in G*erbillus andersoni* (green), *G. pyramidum* (purple), and *G. gerbillus* (red) as a function of host species richness. Lineages found in at least two host individuals are shown in the main figures, and unique lineages are included in the right-hand inserts. Numbers in the right-hand inserts indicate the total number of unique lineages observed for each host species.

The composition of bacterial communities in host individuals was primarily determined by the host species, but also by host diversity and the interaction of the two (Table 2). The SIMPER analysis revealed that *M. haemomuris*-like bacterium (*Mycoplasma* 1 in Fig. 3) was the best lineage for discriminating bacterial communities among individual hosts. In particular, *Mycoplasma* was more prevalent in *G. andersoni* than in the two other host species and in *G. andersoni* single-species plots versus three-species plots. *Bartonella* sp. R4-like bacterium (*Bartonella* 1 in Fig. 3) was the main source of divergence among bacterial communities of *G. andersoni* and *G. pyramidum* individuals, being more prevalent in *G. pyramidum* hosts. A bacterium identified as uncultured *Bifidobacterium* sp. (*Bifidobacterium* 1 in Fig. 3) was the main source of divergence among bacterial communities of *G. andersoni* and *G. gerbillus* hosts, where it was more prevalent in *G. gerbillus* hosts.

### Determinants of bacterial community composition and diversity at the plot scale

#### a) In host populations

Neither host species, host diversity or their interaction had a significant effect on diversity of bacterial communities in host populations sampled at the plot scale (Table 2; Fig. 2B). However, the lineage composition of those communities was determined by the host species (Table 2; Fig. S2 in file S1). SIMPER analysis revealed that five lineages primarily discriminated among bacterial communities in host populations at the plot scale. *M. haemomuris*-like bacterium was more prevalent in *G. andersoni* than in *G. pyramidum* and *G. gerbillus* populations. The uncultured *Bifidobacterium* sp. and *Halomonas phoceae*-like bacterium (*Halomonas* 1 in Fig. 3) was more prevalent in *G. gerbillus* than in *G. andersoni* and *G. pyramidum* populations. Finally, *Bartonella* sp. R4-like bacterium and *Wolbachia* sp. (*Wolbachia* 1 in Fig. 3) were more prevalent in *G. pyramidum* than in *G. gerbillus* populations.

#### b) In host communities

Host diversity did not have a significant effect on lineage diversity or composition of bacterial communities in host communities sampled at the plot scale (Table 2; Fig. 2C)

### Determinants of bacterial community diversity at the regional scale and its relation to potential disease risk

#### a) In host populations

At the regional scale, diversity of bacteria in host populations did not increase, and even showed a decreasing trend, with increased host diversity (Fig. 2D).

#### b) In host communities

As predicted, at the regional scale diversity of bacteria in host communities increased with host diversity (Fig. 2E). In single-host species communities *M. haemomuris*-like bacterium dominated, with almost 80% prevalence. In two-host species communities both *M. haemomuris*-like bacterium and *Bartonella* sp. R4-like bacterium were detected in 50% of the rodent individuals, whereas the prevalence of other lineages were less than 20% (Fig. 3). The most diverse bacterial communities were found in the three-host species plots, with five lineages detected in 24–35% host individuals with no single lineage dominating (Fig. 3).

The model selection approach suggests that the shape of the RSOC changes from an asymmetric sigmoidal curve [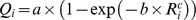; where *Q_i_* is the prevalence of lineage i, *R_i_* is the ranked order of lineage i, and a–c are coefficients] in single-species communities to exponential concave (*Q_i_* = *c*+(*a*×exp(−*b*×*R_i_*)) in multi-species communities (Table 3 and Fig. S3 in file S1). This indicates that bacteria shift from bimodal communities with a peak of common species and another peak of rare species in single-host species communities to a unimodal peak of rare species in multi-species communities. Moreover, the estimated parameters for the symmetric sigmoidal curves of the two multi-species communities were significantly different with a = 0.7±0.06 confidence interval (CI), b = 0.4±0.04 CI, y_0_ = 0.03±0.004 CI for two-species plots and a = 0.4±0.03 CI, b = 0.2±0.02 CI, c = 0.06±0.004 CI, for three-species plots (Fig. S3 in file S1), suggesting that the prevalence of the common species is quantitatively higher in two-species than in three-species communities (Fig.S3 in file S1).

**Table 3:** Results summary for ranked bacterial lineage occupancy analyses for the three rodent communities.

Model/community	Single-species		Two-species		Three-species	
	AIC_c_	w_i_	AIC_c_	w_i_	AIC_c_	w_i_
**Exponential concave**	−155	0.0	**−264**	**1.0**	**−268**	**1.0**
**Exponential convex**	−101	0.0	−172	0.0	−186	0.0
**Lognormal function**	−27	0.0	−44	0.0	−66	0.0
**Sigmodial symmetric**	−135	0.0	−223	0.0	−197	0.0
**Sigmodial asymmetric**	**−169**	**1.0**	−244	0.0	−246	0.0
**Linear regression**	−104	0.0	−174	0.0	−189	0.0

All possible models [41] were analyzed for each rodent community, and the best models as retaining the most information by corrected Akaike information criterion (AIC_c_) are marked in bold. *w_i_* is the Akaike weight of that model, which gives a measure of the plausibility, on a 0 to 1 scale, that a particular model is indeed the best model [60].

#### c) Relationship between host diversity and potential disease risk

13 candidate zoonotic, vector-borne lineages were detected (Table 1). The likelihood of finding these lineages increased with host diversity from 2% (1 out of 53 lineages) in single-species plots, to 5% (4 out of 76 lineages) in two-species plots and 17% (12 out of 72 lineages) in three-species plots, respectively, demonstrating the potential for bacterial pathogens to be more diverse with increasing host diversity (observed data is significantly different from the expected by the differences in the total number of bacterial lineages in one-host species versus three-host species communities; Exact test of goodness of fit, after sequential Bonferroni correction for multiple tests). From the host perspective, the likelihood for a host individual to be infected by at least one candidate zoonotic lineage increased from 11% (4/36) in single-species to 27% (10/37) and 29% (5/17) in two- and three-species plots, although these numbers did not significantly differ from expected after sequential Bonferroni correction for multiple tests. Moreover, the mean number of candidate zoonotic lineages per host individual increased from 0.1±0.05 in single-species to 0.3±0.07, and 0.8±0.5 in two- and three-species plots.

## Discussion

Synthesis of niche theory and island biogeography theory predicts positive associations between habitat heterogeneity and species diversity in communities dominated by organisms with broad niches sampled at large scales [22]. Using a natural field test, we characterized the community structure of bacteria associated with rodent blood and determined the roles of host species and host species richness in shaping bacterial communities at several organizational levels nested in hierarchical spatial scales. Our results demonstrate that bacterial communities are comprised primarily of rare lineages and lineages that can infect more than one rodent species. Despite the uniform sampling efforts and techniques used across plots and host individuals, at fine scales, bacterial diversity and/or composition in individuals and populations of hosts was associated with the host species, whereas at the regional scale bacterial diversity in host communities was positively associated with host species richness. These findings support the theory and have applied implications for predicting overall disease risk, as discussed below.

### Community structure of rodent-associated bacteria

Microbes typically have shorter generation times, larger population sizes, a greater inherent genetic variation and higher rates of migration than vertebrate hosts. Bacteria in vertebrate blood, in addition, are influenced by hormones and the circulating immune response of the hosts. It is therefore expected that microbes in general [44], and bacteria in the blood in particular, will be specifically adapted to their host species. Our study demonstrates that different rodent species are indeed perceived by their bacteria as different habitats. SIMPER analysis demonstrates that the prevalence of bacterial lineages in communities of the three host species is different (Fig. S2 in file S1). Moreover, the addition of *G. gerbillus* to the two-host species communities had divergent effects on lineage diversity of *G. andersoni* versus *G. pyramidum* hosts (Fig. 2A). However, most lineages that occurred in at least three samples were not restricted to a single host species. The ability of bacteria to persist in different gerbil species may be a result of the close phylogenetic distance among the three host species [45], [46], their similar macro-habitat preferences and overlapping geographical distributions [47], their infestation by the same arthropod species [32] and their adaptations to the extreme conditions of the sandy desert [48].

### Association of host and bacterial diversity across spatial scales

The ‘positive host-microbial diversity’ hypothesis predicts that a community dominated by microbes that can infect multiple host species will be less prone to stochastic extinctions and more likely to show a positive response to host diversity, especially when host-bacterial diversity relationships are assessed at large scales. For *M. haemomuris*-like bacterium, which exhibits specificity toward *G. andersoni*, an increase in host diversity was associated with decreased availability of suitable hosts and consequent population reduction (i.e. a ‘dilution effect’: Negative relations between host diversity and disease risk; Fig. 3 [31]). However, it appears that at the regional scale and for the whole bacterial community, the overall effect of decreased availability of suitable hosts with increased host diversity is weaker than the effect of adding new host species, resulting in a positive host-bacterial diversity relationship. In fact, it is possible that the reduction in *M. haemomuris*-like bacterium itself, the most prevalent bacterial lineage, allows rare lineages to invade the bacterial community. The inconsistency between the negative trend of associations between host and bacterial diversity in host populations (Fig. 2D) and the positive trend in host communities (Figs. 2E, 3, S3 in file S1) suggests that the increase in bacterial diversity in multi-species host communities results from variation among host species.

At the regional scale, increasing host diversity had dual effects on diversity of the bacterial community. On one hand, the total number of bacterial lineages increased with host diversity, as evidenced by the length of the tail of the three RSOC curves. Note that the length of the tail of the three-host species curve was almost as long as that of the two host curves, despite a large difference in the total number of host individuals sampled (17 and 37 respectively). The increase in bacterial richness with host species richness may be attributed to the addition of more bacterial lineages specialized on the new host species, which have lower stochastic extinction rates at the regional scale. On the other hand, the prevalence of the bacterial lineages that are most common in single-host species communities decreased with the increase in host diversity. This decrease is associated with a change from bacterial lineages with narrow niche breadth to lineages with a wider niche breadth as evident in Fig. 3. The overall prevalence of the latter bacterial lineages may be lower since they trade off performance for generality. Taken together, when host species richness increases, the bacterial community is dominated by generalist lineages (but with lower prevalence) accompanied by a long tail of specialist species, while under low host species richness, the common species are specialists with a shorter tail of other specialist and some generalist species. Future research should test whether similar relationships exist between host species richness and the structure of other bacterial communities that are more prone to environmental influences such as bacteria in the gut, oral cavity, and skin.

We are unable to determine whether host species richness is the driver for the changes in microbial diversity due to the observational nature of the study. However, it is unlikely that the observed host-microbial diversity relationships are indirect given the similar macro-habitats of the three types of host communities and the nature of the sampled bacteria which are likely to be affected mainly by host traits and not by the external environment.

Broad bacterial niches should not be restricted to communities of closely-related host species or to blood microbiota. The ability of common species to utilize multiple habitats or hosts has been documented for free-living macro-organisms and microbes as well as for macro-parasites [1], [2], [49]. This ability of common species of various organisms in natural communities to utilize more than one habitat or host species may decrease their likelihood of stochastic extinction, especially at large spatial scales, explaining the widespread occurrence of positive relationships between habitat heterogeneity and species diversity [1]–[5].

### Applied perspective

There is considerable interest in how communities of hosts and their associated microorganisms assemble in nature based on the hypothesis that community structure affects the transmission rates of pathogens among host individuals [10]. Recent work on amphibians suggests that their community composition changes consistently along a gradient of species richness, leading to a nested pattern in which low-diversity communities formed near-perfect subsets of more diverse assemblages [6]. Because low-diversity communities are also dominated by highly competent hosts for the virulent pathogen *Ribeiroia ondatrae*, this community structure leads to a ‘dilution effect’ [6]. The natural rodent communities in the Negev desert also form a nested subset pattern. Likewise, *M. haemomuris*-like bacterium is diluted in more diverse host species communities. However, since bacteria within those gerbil communities comprise mostly rare lineages and lineages that likely do not cause vertebrate diseases (Fig. 3 & Table S1 in file S1), the nested pattern of gerbil communities does not lead to an overall ‘dilution effect’ and may instead lead to a positive relationship between host diversity and disease risk. The positive effect results from the positive relationship between host and bacterial species diversity and the associated increased likelihood of rare pathogens to be found in diverse bacterial communities. The discrepancy between the two studies suggests that, in addition to the community structure of hosts, the community structure of microbes should be considered when predicting disease risk. In this study we focused solely on the occurrence pattern of microbes in the community, but disease risk might be increased further due to positive interactions among microbes in the community [10].

It is suggested that the ‘dilution effect’ is a far more common consequence of high host diversity than the opposite ‘amplification effect’ [50]. Based on the dilution effect, it is often proposed that management actions aimed at reducing disease risk to humans should increase host species diversity [51]. Yet most tests of the dilution hypothesis have focused on the changes in prevalence of single disease-causing agents at a time, with no attention to the relations between host diversity and microbial diversity. Our findings demonstrate that while such decisions are applicable to areas that are threatened mainly by a pathogen with a relatively narrow competent-host range (e.g., Lyme disease in the eastern United States), they may lead to unintended results in other areas that are threatened by the emergence of rare pathogens. In the latter areas, a rise in host diversity may result in a greater diversity of rare pathogens and a higher risk of infection by them.

In summary, the results presented here broaden our understanding of how communities of hosts and their associated microbes are structured in nature, and how microbial diversity is affected by host diversity at different spatial scales and levels of organization. Our study highlights the importance of taking a community approach since the full microbial response to host biodiversity, including those of rare pathogens, may be different from responses by a single pathogen.

## Supporting Information

File S1 **Supporting information: Methods, tables, and figures.** Supplement Methods, Detailed description of methods including the procedure of blood sampling, the protocols of PCR and sequencing and statistical analysis. Table S1, Average abundance of the different genera in blood samples of the three host species. Figure S1, The distribution of the numbers of bacterial lineages occupying different numbers of host individuals. Bacterial classifications from known sequences that best matched the sample sequence are provided for the three most prevalent lineages (i.e., lineages that occur in 16 or more host individuals). Figure S2, Non-metric multidimensional scaling (MDS) ordination of blood samples based on Bray-Curtis similarities in the presence/absence data of the lineages in bacterial communities of rodent populations separates samples by host species. Each point represents the bacterial community in a given host species in a plot; *Gerbillus andersoni* individuals are indicated in green, *G. pyramidum* in purple and *G. gerbillus* in red. Point proximities represent the extent of similarity in bacterial community compositions. Oval shapes surround similar bacterial communities in host populations of the same species. Note that for an improved illustration, one outlier was omitted from the figure (bacterial community in one population of *G. gerbillus*). Figure S3, Ranked bacterial lineage occupancy ( = prevalence) curves in the three types of rodent communities: single-rodent species (red), two-rodent species (light blue), and three rodent species (purple) communities. Best fitted curves are indicated by lines.
(DOCX)
