# Supplementary material for: Association of Host and Microbial Species Diversity across Spatial Scales in Desert Rodent Communities
Source: PLoS One. 2014 Oct 24;9(10):e109677. doi: 10.1371/journal.pone.0109677 (PMC4208758; doi:10.1371/journal.pone.0109677)
Supplement: File S1 — Supporting information: Methods, tables, and figures. Supplement Methods, Detailed description of methods including the procedure of blood sampling, the protocols of PCR and sequencing and statistical analysis. Table S1, Average abundance of the different genera in blood samples of the three host species. Figure S1, The distribution of the numbers of bacterial lineages occupying different numbers of host individuals. Bacterial classifications from known sequences that best matched the sample sequence are provided for the three most prevalent lineages (i.e., lineages that occur in 16 or more host individuals). Figure S2, Non-metric multidimensional scaling (MDS) ordination of blood samples based on Bray-Curtis similarities in the presence/absence data of the lineages in bacterial communities of rodent populations separates samples by host species. Each point represents the bacterial community in a given host species in a plot; Gerbillus andersoni individuals are indicated in green, G. pyramidum in purple and G. gerbillus in red. Point proximities represent the extent of similarity in bacterial community compositions. Oval shapes surround similar bacterial communities in host populations of the same species. Note that for an improved illustration, one outlier was omitted from the figure (bacterial community in one population of G. gerbillus). Figure S3, Ranked bacterial lineage occupancy ( = prevalence) curves in the three types of rodent communities: single-rodent species (red), two-rodent species (light blue), and three rodent species (purple) communities. Best fitted curves are indicated by lines. (DOCX) [file pone.0109677.s001.docx]

**File S1: Supporting information; Methods, Tables and Figures**

**Supplement Methods**

**Blood sampling**

Blood samples were drawn from the orbital sinus of the eye under local anesthesia using Localin (0.4 % benoxinate hydrochloride) (Fisher Pharmaceutical Labs, Tel Aviv, Israel). Using heparinized capillaries, the blood was collected into and stored in EDTA-containing tubes at –20°C. Due to the sensitivity of the eyes we could not sterilize the local area before bleeding. To minimize contamination from external surfaces of the eye, we kept the bleeding process as quick as possible and frequently changed the capillaries. These efforts cannot absolutely prevent possible surface contamination. However, since all host individuals were subjected to the exact sampling procedure, possible surface contaminants should only increase the variance among the composition of bacterial communities leading to the inflation of type II error, and should not bias the results toward a positive or a negative relationship between host and bacterial diversity.

**PCR and sequencing**

V1-V3 region 16S rRNA PCR reactions included 2-10 µl of blood gDNA preparation, Phusion high fidelity DNA polymerase (New England Biolabs, Ipswich, MA) and oligonucleotide primers 27F, which additionally contained an adaptor sequence B, and 534R coupled to the A adaptor sequence and a unique barcode (454 Life Sciences, Branford CT). PCR amplicons were purified by a Qiaquick gel extraction kit (Qiagen) and quantified by Quant-It HS double stranded DNA assay (Invitrogen, Carlsbad CA). The individual samples were then mixed together in equimolar concentrations. Emulsion PCR and 454 library generation steps were performed according to the manufacturer’s instructions (454 Life Sciences). 454 sequencing was performed on a Roche/454 GS-FLX Titanium system at the Indiana University Center for Genomics and Bioinformatics, Bloomington IN.

**Statistical analysis**

***Resampling procedure:*** Lineage composition could have been affected by the total number of sequences, given that the total number of sequences per sample ranged from 35 to 353 sequences, with a mean of 161 and a mode of 64. Therefore, we normalized the number of sequences in each sample by randomly selecting 35 sequences from each sample 1000 times (re-sampling with replacement) and used the average number of sequences detected for each lineage-sample combination in the ‘lineage composition’ analyses.

***Quantification of bacterial lineage diversity and composition:*** We explored the effects of the host species, host diversity and their interaction (independent variables) for each combination of organization level and scale, on the diversity (Fisher’s alpha) and composition (binary Bray-Curtis) of bacterial lineages (dependent variables). Effects on lineage diversity were analyzed by Generalized Linear Models (GLM), assuming gamma distribution with a log-linked function. Effects on lineage composition were analyzed by non-parametric multivariate multiple regression, with a stepwise forward-selection procedure, using the computer program DISTLM [[1](#_ENREF_1)]. To visualize patterns in multivariate data, an unconstrained non-metric multi-dimensional scaling (MDS) plot was used, based on Bray-Curtis similarities on the presence/absence transformed assemblage data (using the program PRIMER-E).

***Quantifications of Ranked bacterial lineage (Species) Occupancy Curves (RSOC):*** To complement the qualitative presentation of host diversity on bacterial diversity at the regional scale, we performed non-linear regressions of RSOC and competed among six regression models, following Jenkins [[2](#_ENREF_2)]. In short, using a model selection approach, we competed among six regression models, which are used to characterize the rank occupancy relationships of most ecological communities ([Table 1 in reference 2](#_ENREF_2)). Because these models have different numbers of parameters, Akaike information criterion (corrected for small sample sizes; AIC_c_) was used to select the best model fitting the empirical data ([3; Table 3](#_ENREF_3)). The model with the lowest AIC_c_ was assigned as the form of RSOC to a specific rodent community.

**Table S1:** Average abundance of the different genera in blood samples of the three host species

| **Genus or *higher taxonomic affiliation** | ***G. andersoni*** | ***G. pyramidum*** | ***G. gerbillus*** |
| --- | --- | --- | --- |
| Acidobacteria CLASS | 0.0E+00 | 0.0E+00 | 4.3E-02 |
| Acidobacteriaceae FAMILY | 0.0E+00 | 0.0E+00 | 4.3E-02 |
| *Acidovorax* | 3.3E-02 | 0.0E+00 | 0.0E+00 |
| *Adhaeribacter* | 0.0E+00 | 0.0E+00 | 8.7E-02 |
| *Afipia* | 2.5E-02 | 0.0E+00 | 0.0E+00 |
| *Agromonas* | 5.0E-02 | 0.0E+00 | 0.0E+00 |
| *Arthrobacter* | 2.0E-01 | 0.0E+00 | 1.3E-01 |
| Bacillales ORDER | 7.0E-01 | 0.0E+00 | 0.0E+00 |
| *Bacillus* | 2.9E-02 | 0.0E+00 | 1.1E-02 |
| Bacteroidales ORDER | 0.0E+00 | 0.0E+00 | 8.7E-02 |
| *Balneimonas* | 0.0E+00 | 0.0E+00 | 4.3E-02 |
| *Bartonella* | 4.2E+00 | 5.9E+00 | 1.8E+01 |
| *Blastochloris* | 1.7E-02 | 0.0E+00 | 0.0E+00 |
| *Blastococcus* | 5.0E-02 | 4.3E-01 | 4.3E-02 |
| *Bosea* | 1.7E-02 | 0.0E+00 | 0.0E+00 |
| *Bradyrhizobium* | 3.7E-01 | 0.0E+00 | 2.9E-01 |
| *Capnocytophaga* | 8.2E-01 | 1.9E+00 | 6.7E-01 |
| *Cardinium* | 0.0E+00 | 0.0E+00 | 5.7E-01 |
| *Cloacibacterium* | 1.1E+00 | 0.0E+00 | 1.8E+00 |
| Clostridia CLASS | 0.0E+00 | 2.1E-01 | 2.2E-02 |
| *Clostridium* | 0.0E+00 | 0.0E+00 | 1.7E+00 |
| Comamonadaceae FAMILY | 1.7E-02 | 0.0E+00 | 0.0E+00 |
| *Comamonas* | 1.7E-02 | 0.0E+00 | 0.0E+00 |
| *Corynebacterium* | 3.3E-03 | 0.0E+00 | 3.5E-02 |
| *Coxiella* | 1.2E+00 | 1.1E+01 | 0.0E+00 |
| *Deinococcus* | 2.5E-02 | 0.0E+00 | 0.0E+00 |
| *Diaphorobacter* | 5.2E+00 | 0.0E+00 | 0.0E+00 |
| *Eubacterium* | 0.0E+00 | 0.0E+00 | 7.0E-01 |
| *Finegoldia* | 0.0E+00 | 4.3E-01 | 0.0E+00 |
| Firmicutes PHYLUM | 6.0E-01 | 2.4E+00 | 0.0E+00 |
| Gemmatimonadetes FAMILY | 5.0E-02 | 0.0E+00 | 0.0E+00 |
| *Halomonas* | 5.9E-01 | 1.7E+00 | 5.2E-01 |
| *Janthinobacterium* | 0.0E+00 | 0.0E+00 | 4.3E-02 |
| *Kineosporia* | 3.3E-02 | 0.0E+00 | 0.0E+00 |
| *Kocuria* | 1.0E-01 | 0.0E+00 | 0.0E+00 |
| *Lactobacillus* | 0.0E+00 | 6.4E-01 | 2.2E-02 |
| *Leptotrichia* | 0.0E+00 | 0.0E+00 | 8.7E-02 |
| *Luteimonas* | 1.7E-02 | 0.0E+00 | 0.0E+00 |
| *Massilia* | 1.1E-01 | 0.0E+00 | 0.0E+00 |
| *Mesorhizobium* | 1.7E-02 | 0.0E+00 | 0.0E+00 |
| *Methylobacterium* | 2.0E-01 | 3.4E-01 | 8.7E-02 |
| *Micrococcus* | 5.0E-02 | 0.0E+00 | 0.0E+00 |
| *Microlunatus* | 0.0E+00 | 0.0E+00 | 4.3E-02 |
| *Mycoplasma* | 2.6E+01 | 3.3E-01 | 2.0E-01 |
| *Naxibacter* | 7.2E-02 | 0.0E+00 | 8.7E-02 |
| *Neisseria* | 5.0E-02 | 0.0E+00 | 0.0E+00 |
| *Nocardioides* | 0.0E+00 | 4.3E-01 | 0.0E+00 |
| *Novosphingobium* | 5.0E-02 | 0.0E+00 | 0.0E+00 |
| *Pedomicrobium* | 3.3E-02 | 0.0E+00 | 0.0E+00 |
| *Pelomonas* | 1.7E-01 | 2.9E-01 | 0.0E+00 |
| *Planococcus* | 6.7E-02 | 4.3E-01 | 0.0E+00 |
| *Planomicrobium* | 1.8E-01 | 0.0E+00 | 0.0E+00 |
| *Pontibacter* | 0.0E+00 | 0.0E+00 | 4.3E-02 |
| *Porphyrobacter* | 5.0E-02 | 0.0E+00 | 0.0E+00 |
| *Porphyromonas* | 0.0E+00 | 5.7E-01 | 0.0E+00 |
| *Propionibacterium* | 1.6E-01 | 2.3E+00 | 8.7E-02 |
| *Pseudonocardia* | 5.8E-02 | 0.0E+00 | 0.0E+00 |
| *Ralstonia* | 6.7E-02 | 0.0E+00 | 1.5E-01 |
| *Rhizobium* | 1.7E-02 | 0.0E+00 | 0.0E+00 |
| *Rhodocytophaga* | 3.3E-02 | 0.0E+00 | 0.0E+00 |
| *Roseococcus* | 0.0E+00 | 0.0E+00 | 8.7E-02 |
| *Rothia* | 0.0E+00 | 0.0E+00 | 8.7E-02 |
| *Rubellimicrobium* | 4.2E-02 | 0.0E+00 | 2.2E-02 |
| *Rubrobacter* | 0.0E+00 | 0.0E+00 | 4.3E-02 |
| *Saccharothrix* | 3.8E-01 | 0.0E+00 | 0.0E+00 |
| *Sediminibacterium* | 1.0E-01 | 0.0E+00 | 0.0E+00 |
| *Shewanella* | 1.9E-01 | 0.0E+00 | 0.0E+00 |
| *Sphaerobacter* | 0.0E+00 | 0.0E+00 | 4.3E-02 |
| *Sphingobacteria CLASS* | 2.2E-01 | 0.0E+00 | 0.0E+00 |
| *Sphingomonas* | 4.4E-02 | 0.0E+00 | 5.8E-02 |
| *Spiroplasma* | 2.3E-01 | 0.0E+00 | 4.3E-02 |
| *Staphylococcus* | 2.9E-01 | 0.0E+00 | 0.0E+00 |
| *Streptococcus* | 2.2E-01 | 8.6E-01 | 0.0E+00 |
| *Streptomyces* | 1.7E-02 | 1.4E-01 | 6.5E-02 |
| Thermoanaerobacterales FAMILY | 0.0E+00 | 4.3E-01 | 0.0E+00 |
| *Thermomicrobium* | 3.3E-02 | 0.0E+00 | 0.0E+00 |
| TM7 PHYLUM | 0.0E+00 | 0.0E+00 | 8.7E-02 |
| *Trichococcus* | 3.3E-01 | 0.0E+00 | 0.0E+00 |
| Uncultured endolithic bacterium | 3.3E-02 | 0.0E+00 | 0.0E+00 |
| *Veillonella* | 0.0E+00 | 0.0E+00 | 1.2E-01 |
| *Wolbachia* | 4.9E-01 | 4.9E-01 | 8.2E-01 |
| *Xanthobacter* | 1.7E-02 | 0.0E+00 | 0.0E+00 |
| *Xanthomonas* | 1.7E-02 | 0.0E+00 | 0.0E+00 |
| *Rickettsia* | 3.5E+00 | 0.0E+00 | 0.0E+00 |
| *Azovibrio* | 0.0E+00 | 0.0E+00 | 1.3E-01 |
| Armatimonadetes PHYLUM | 3.3E-02 | 0.0E+00 | 0.0E+00 |
| Rhizobiales ORDER | 1.7E-02 | 0.0E+00 | 0.0E+00 |
| Clostridiales ORDER | 0.0E+00 | 0.0E+00 | 8.7E-02 |
| Cyanobacterium PHYLUM | 1.2E-01 | 0.0E+00 | 6.5E-02 |
| Proteobacterium PHYLUM | 0.0E+00 | 0.0E+00 | 4.3E-02 |
| Streptophyta PHYLUM | 0.0E+00 | 4.3E-01 | 0.0E+00 |

***** When the genus could not be determined, a higher taxonomic affiliation is given

**Fig. S1** The distribution of the numbers of bacterial lineages occupying different numbers of host individuals. Bacterial classifications from known sequences that best matched the sample sequence are provided for the three most prevalent lineages (i.e., lineages that occur in 16 or more host individuals).

**Fig. S2.** Non-metric multidimensional scaling (MDS) ordination of blood samples based on Bray-Curtis similarities in the presence/absence data of the lineages in bacterial communities of rodent populations separates samples by host species. Each point represents the bacterial community in a given host species in a plot; *Gerbillus andersoni* individuals are indicated in green, *G. pyramidum* in purple and *G. gerbillus* in red. Point proximities represent the extent of similarity in bacterial community compositions. Oval shapes surround similar bacterial communities in host populations of the same species. Note that for an improved illustration, one outlier was omitted from the figure (bacterial community in one population of *G. gerbillus).*

**Fig. S3.** Ranked bacterial lineage occupancy (=prevalence) curves in the three types of rodent communities: single-rodent species (red), two-rodent species (light blue), and three rodent species (purple) communities. Best fitted curves are indicated by lines.

**References**

1. McArdle BH, Anderson MJ (2001) Fitting multivariate models to community data: a comment on distance-based redundancy analysis. Ecology 82: 290-297.

2. Jenkins DG (2011) Ranked species occupancy curves reveal common patterns among diverse metacommunities. Global Ecology and Biogeography 20: 486-497.

3. Burnham KP, Anderson DR (2002) Model Selection and Multimodel Inference. A Practical Information-Theoretic Approach. New York: Springer Science and Business Media.
